# The Potential Protective Action of Vitamin D in Hepatic Insulin Resistance and Pancreatic Islet Dysfunction in Type 2 Diabetes Mellitus

**DOI:** 10.3390/nu8030147

**Published:** 2016-03-05

**Authors:** Po Sing Leung

**Affiliations:** School of Biomedical Sciences, Faculty of Medicine, The Chinese University of Hong Kong, Shatin, Hong Kong, China; psleung@cuhk.edu.hk; Tel.: +852-3943-6879 (ext. 36879); Fax: +852-2603-5139

**Keywords:** calcitriol, glucose homeostasis, HepG2, hypovitaminosis D, insulin secretion, lipid metabolism

## Abstract

Vitamin D deficiency (*i.e.*, hypovitaminosis D) is associated with increased insulin resistance, impaired insulin secretion, and poorly controlled glucose homeostasis, and thus is correlated with the risk of metabolic diseases, including type 2 diabetes mellitus (T2DM). The liver plays key roles in glucose and lipid metabolism, and its dysregulation leads to abnormalities in hepatic glucose output and triglyceride accumulation. Meanwhile, the pancreatic islets are constituted in large part by insulin-secreting β cells. Consequently, islet dysfunction, such as occurs in T2DM, produces hyperglycemia. In this review, we provide a critical appraisal of the modulatory actions of vitamin D in hepatic insulin sensitivity and islet insulin secretion, and we discuss the potential roles of a local vitamin D signaling in regulating hepatic and pancreatic islet functions. This information provides a scientific basis for establishing the benefits of the maintenance, or dietary manipulation, of adequate vitamin D status in the prevention and management of obesity-induced T2DM and non-alcoholic fatty liver disease.

## 1. Introduction

The liver is a vital organ for metabolic homeostasis, controlling glucose uptake-storage-generation and processing about one third of consumed glucose, and it is a key target for insulin action [1]. Insulin controls lipogenesis (fatty acid and triglycerides biosynthesis) and restrains hepatic gluconeogenesis (glucose production) in the liver. Accordingly, insulin sensitivity has been closely associated with rates of hepatic gluconeogenesis and lipid accumulation [2,3]. Hepatic insulin resistance leads to severely dysregulated glucose homeostasis, resulting in or contributing to hyperglycemia, which then further worsens hepatic insulin insensitivity. This negative cycle progresses toward a pathologic state of hepatic dysfunction and eventually liver disease. Notwithstanding these observations, it is unclear whether hepatic insulin resistance is a cause or consequence of type 2 diabetes mellitus (T2DM), and the underlying mechanism(s) mediating the relationship between hepatic insulin resistance and T2DM remain elusive. In addition to conventional regulatory factors (e.g., genes encoding glycolytic and lipogenic enzymes), changes in life style and nutritional factors have garnered ever increasing attention in the development of preventative and therapeutic approaches to both T2DM and non-alcoholic fatty liver disease (NAFLD). In this context, recent investigations into the interactions of nutritional factors, such as vitamin D, in T2DM-related diseases and their management are of particular interest in terms of both clinical and socio-economic impacts [4,5,6].

The endocrine pancreas (functionally distinct from the exocrine portions important for production of digestive enzymes) contains insulin-secreting pancreatic islets [7]. Dysfunction or loss of beta cells in pancreatic islets leads to hyperglycemia and hyperlipidemia, two predominant indicators of T2DM [8]. Islet physiology is finely modulated by a myriad of factors, including vitamin D. Vitamin D deficiency, referred to clinically as hypovitaminosis D, impairs insulin secretion and increases insulin resistance, key features of T2DM and related metabolic diseases [9,10]. Interestingly, glucose-stimulated insulin response experiments suggest that early vitamin D supplementation may be protective of insulin secretory function, whereas late supplementation, after vitamin D deficiency and T2DM have been established, may be relatively ineffective [11]. However, the precise modulatory roles of vitamin D in hepatic and islet functions have not been determined.

The purpose of the present review is to provide a critical appraisal of our current knowledge related to the protective effects of vitamin D on hepatic metabolism and islet function, particularly in the context of T2DM and obesity-associated diseases. Such information will be important for informing healthcare providers and patients about the potential benefits of vitamin D supplementation, as a food additive or nutraceutical. In particular, this work focuses on the potential of vitamin D supplementation to oppose the development or worsening of obesity-related diseases, such as T2DM and NAFLD, and thus provide a cost-effective measure for improving hepatic and pancreatic islet functions.

## 2. Vitamin D Synthesis and Metabolism

The pathophysiology of the insulin-resistant state and islet dysfunction remains enigmatic. Currently, patients with insulin resistance and islet dysfunction are treated with insulin sensitizers and secretagogues, which work mainly by improving glucose disposal via skeletal muscle and suppressing hepatic gluconeogenesis, and by stimulating pancreatic beta-cell insulin secretion, respectively [12]. Mechanism-based studies of the factors that influence insulin secretion and resistance, such as niacin [13,14] and vitamin D (*vide infra*), are needed to identify novel therapeutic targets.

Classically, vitamin D is known for its involvement in the regulation of calcium and bone homeostasis. There are two major forms of vitamin D, namely cholecalciferol (vitamin D_3_) and ergocalciferol (vitamin D_2_). The primary source of vitamin D in humans is from endogenous biosynthesis in skin cells, a critical step of which hydroxylation of 7-dehydrocholesterol into cholecalciferol is catalyzed by ultraviolet radiation from sunlight [15]. Additionally, cholecalciferol can be found in dietary sources, including fish, egg yolk, beef, and lichen, while ergocalciferol can be found in edible mushrooms and alfalfa. Endogenous and dietary vitamin D molecules, which are biologically inactive themselves, are transported systemically in chylomicrons and converted into the metabolically active 1,25-dihydroxyvitamin D (1,25(OH)_2_D_3_) via a sequence of reactions. In the liver, vitamin D is converted into 25-hydroxyvitamin D (25(OH)D_3_), the clinically monitored form of plasma vitamin D, by the liver-derived enzyme 25-hydroxylase. Subsequently, 25(OH)D_3_, which is considered a prehormone, is then further hydroxylated to biologically active 1,25(OH)_2_D_3_ by kidney-derived 1-α-hydroxylase [16].

**Figure 1:**
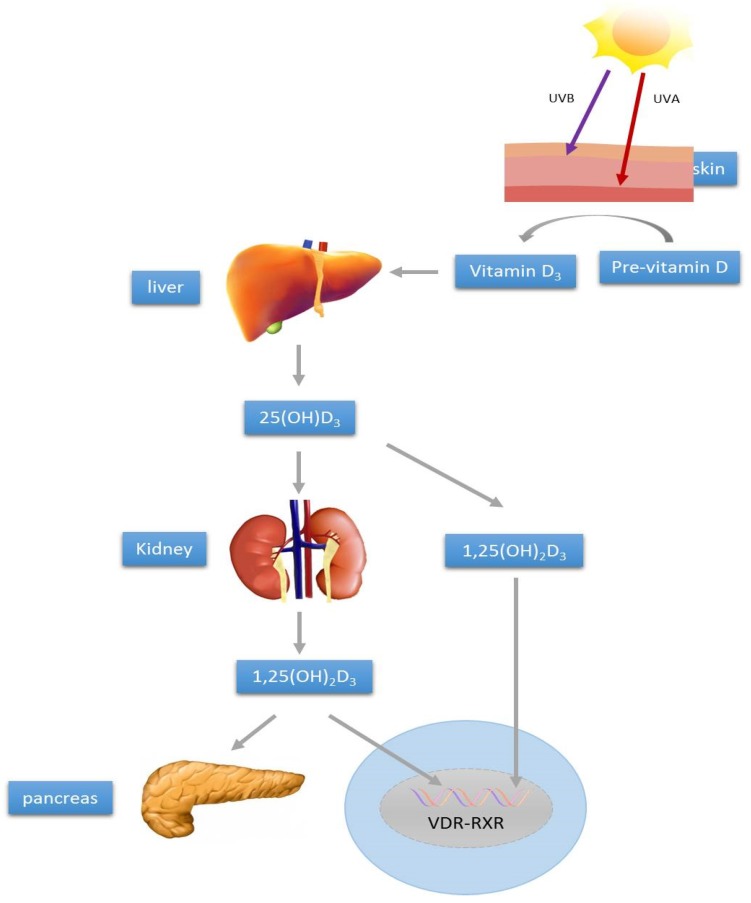
Schematic representation of vitamin D synthesis and metabolism in relation to regulation of pancreatic islet function and survival.

The active form of vitamin D, 1,25(OH)_2_D_3_, binds vitamin D receptors (VDRs) and activates various transcription factors. For example, activated VDR forms a heterodimer with retinoic X receptor (RXR), and thereby participates in stimulating the RXR nuclear pathway [17]. Indeed, classical vitamin D response elements and other response sites are broadly distributed and critical for pancreatic beta-cell and immune system functions [18,19,20,21]. Such findings suggest that manipulations of VDR signaling pathways might be physiologically relevant for the management of diabetes. Figure 1 is a summary of vitamin D synthesis and metabolism, as well as the modulatory action on islet function.

## 3. Vitamin D and T2DM

Hypovitaminosis D has been correlated with increased risks of a wide variety of chronic diseases. The major causes of hypovitaminosis D are sunlight deprivation (such as by sunscreen, melanin, latitude, and winter), medications and supplements, and malabsorption (such as in Crohn’s and celiac diseases, cystic fibrosis, and liver disease). Maintenance of plasma 25(OH)D_3_ above 30 ng/mL is recommended for optimal health; some may require dietary supplementation and/or increased exposure to sunlight to attain this level [15]. Of great interest for our present focus, hypovitaminosis D has been reported to impair islet insulin secretion and to increase peripheral insulin resistance, two major risk factors for progression to T2DM; furthermore, hypovitaminosis D is predictive of abnormalities in most of the variables monitored in patients with metabolic syndromes, including T2DM itself [22,23]. It is worthwhile emphasizing that the clinical association between hypovitaminosis and insulin resistance/T2DM might not be a cause-and-effect relationship and that an increase in circulating 25(OH)D_3_ concentration might not necessarily reduce the onset of, or progression to, T2DM. In this context, there are considerable randomized clinical trials showing disassociation between plasma levels of active 25(OH)D_3_ and the incidence of T2DM, as recently exemplified by a mendelian randomization-derived estimates for glycemic control [24]. In fact, maintenance of adequate vitamin D status and/or its high-dose supplementation have not convincingly displayed long-term glycemic control for human T2DM in several clinical trial studies [25,26,27,28]. Such negative studies or discrepancies have yet to be validated by further large-scale clinical studies with large sample sizes, different study designs (cross-sectional and interventional studies, and different populations), as well as by intensive mechanism-driven basic science studies in the future.

T2DM, which is defined as hyperglycemia of sufficient magnitude to cause detrimental effects, results when insulin resistance develops and is followed by dysregulation of insulin secretory responses, with loss of beta-cell mass [29]. Amelioration of both insulin resistance and islet dysfunction are, therefore, both of paramount importance for preventing and treating T2DM. Chronic hyperglycemia and hyperlipidemia are associated with dramatic upregulation of lipid formation and accumulation in the liver and pancreatic islets. Excessive hepatic lipid accumulation causes endoplasmic reticulum (ER) stress and inflammation (lipotoxicity), as well as reduced insulin sensitivity [30,31]. Meanwhile, an increase in intra-islet lipid accumulation impairs glucose-stimulated insulin secretion, and promotes islet inflammation and ER stress, ultimately leading to beta-cell apoptosis and islet failure [32].

Given the importance of vitamin D status for the maintenance of a physiologically healthy liver and pancreas, together with the known involvement of hepatic and pancreatic pathophysiology in T2DM pathogenesis, vitamin D and the VDR represent an area of great therapeutic interest. Identification of agents that can reduce abnormalities of hepatic and islet metabolism simultaneously could yield a substantial advancement in the prevention and treatment of obesity and obesity-related T2DM risk. To this end, there has been a surge of interest in the benefits of maintaining an adequate vitamin D status and the apparent protective mechanism(s) of vitamin D.

## 4. Vitamin D and Hepatic Metabolism

The liver is a vital organ consisting of functional units called liver lobules, which in turn are composed principally of hepatic cellular plates. The major cell types of the liver are hepatocytes, stellate cells, Kupffer cells, and endothelial cells, with hepatocytes being the functionally predominant cells [1]. As mentioned above, the liver is a major target organ of insulin, wherein insulin regulation helps to maintain glucose homeostasis by making glucose available when it is needed through gluconeogenesis and glycogenolysis, and by storing glucose through glycogenesis when it is present in excess [1]. Gluconeogenesis rate is determined primarily by the transcriptional level of the genes encoding two gluconeogenic enzymes, namely phosphoenolpyruvate carboxykinase (PEPCK) and glucose-6-phosphatase (G6PK) [33]. The opposing process of glycogenesis is mediated by glycogen synthase (GS); GS activity is promoted when glycogen synthase kinase 3 (GSK-3) activity is inhibited [34].

Glucose and lipid metabolism are critical inter-related components of glucose homeostasis. When glucose intake exceeds storage and oxidation capacities, it is converted to fat (*de novo* lipogenesis); however, excessive hepatic lipid causes inflammation and hepatic insulin resistance [35]. Interestingly, numerous studies have suggested that ER stress, which is caused by an imbalance between protein folding stress and the processing capacity of the ER, is closely associated with metabolic disorders. In particular, hepatic ER stress promotes hepatic glucose production, lipogenesis, and insulin resistance in obese and diabetic states [36]. Hence, maintenance of normal hepatic cellular metabolic function appears to be indispensable for preventing the development of hepatic insulin resistance and T2DM.

The metabolic regulating enzyme AMP-activated protein kinase (AMPK) is the therapeutic target of several anti-diabetic agents, such as metformin [37] and adiponectin [38]. It is activated by phosphorylation via either the serine/threonine kinase 11 (a.k.a. liver kinase B1), or the calcium/calmodulin protein kinase kinase beta (CaMKKβ) pathway [39]. The anti-diabetic actions of hepatic AMPK activation are attributed to attenuation of lipogenesis and gluconeogenesis, as well as promotion of lipid oxidation and glycolysis [40]. Additionally, activation of hepatic AMPK has been reported to inhibit Foxo1 activity [41], which results in reduced hepatic ER stress, and to alleviate hepatic steatosis and insulin resistance [42,43]. Furthermore, prior clinical studies have shown that low serum concentrations of 25(OH)D_3_ are independently associated with liver steatosis [44]. Meanwhile, hypovitaminosis D has been proposed to be a causative factor of NAFLD [45]. Although liver steatosis is related to progression of hepatic insulin resistance, no specific lipid has been reported to be both necessary and sufficient for development of liver steatosis. NAFLD appears to involve the accumulation of a variety of lipids, and the levels of multiple lipids can be used as markers of insulin resistance status [46]. Excessive accumulation of certain lipids, such as diacylglycerol and acyl CoA, may interfere with glucose generation and has been linked to risk of hepatic insulin resistance [46,47,48,49]. Because hepatic glucose production is tightly regulated by the availability of the enzymes PEPCK and G6PK, and further modulated by the availability of fructose-1,6-bisphosphatase and pyruvate carboxylase [50], down-regulation of these enzymes might reduce abnormal gluconeogenesis in T2DM and ameliorate hepatic insulin resistance.

The research summarized above has led us to propose that vitamin D bioavailability may affect hepatic lipogenesis and gluconeogenesis, and if so, vitamin D supplementation may be used to modulate hepatic insulin resistance and thus reduce T2DM severity. Mechanistically, such effects may be mediated by various vitamin d-regulated pathways, such as AMPK-calmodulin and/or Akt/Notch signaling, as well as through indirect effects on ER stress. In light of the aforementioned negative association between vitamin D status and severity of NAFLD, a well-recognized risk factor for insulin resistance and T2DM, we are now investigating the direct effects of vitamin D on hepatic lipid and glucose production. We obtained preliminary data recently indicating that, at high dosages, calcitriol (the active hormonal metabolite of vitamin D) can ameliorate abnormal hepatic lipid and glucose metabolism in both *in vitro* (1–10 nM in HepG2 cells) and *in vivo* (0.5–2.5 mg/kg for 2 days in db/db mice) models of insulin resistance without any signs of toxicity (unpublished data). Furthermore, we conducted mechanistic experiments showing that increases in cytosolic calcitriol in HepG2 cells activated Ca^2+^/CaMKKβ/AMPK pathways, and that the activation of these pathways contributed to calcitriol’s lipid and glucose regulatory effects.

**Figure 2:**
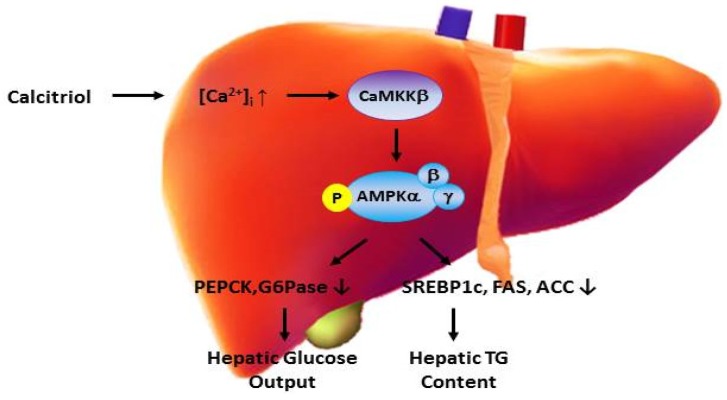
Model of active vitamin D regulation of hepatic triglyceride accumulation and glucose output in a diabetic state.

The involvement of AMPK signaling in calcitriol-mediated metabolic effects is not surprising given that AMPK is the therapeutic target of anti-diabetic medications (e.g., metformin) [37], as well as a target of the obesity regulating endogenous hormone adiponectin, which acts to alleviate insulin resistance [38]. Further study is needed to corroborate these findings, which suggest that calcitriol, when at above-physiological plasma concentrations, can reduce hepatic triglyceride accumulation and glucose output, at least in part, through activation of Ca^2+^/CaMKKβ/AMPK signaling under insulin-resistant conditions. Importantly, unlike its inactive metabolic precursor cholecalciferol (vitamin D_3_), calcitriol does not accumulate in adipose tissue or exert long-lasting effects. Thus, the potential for toxicity that exists with cholecalciferol is much less of a concern with calcitriol, owing to its rapid onset and offset of action. If confirmed, these preliminary data may provide an avenue to supporting the use of vitamin D, at least, as an adjuvant for the management of insulin resistance, NAFLD, and T2DM. Figure 2 is a summary that proposes the direct action of vitamin D in regulating hepatic triglyceride and glucose metabolism.

## 5. Vitamin D and Pancreatic Islet Function

Emerging data from physiological and genetic studies indicate that islet dysfunction and loss of beta-cell mass are the key determinants of whether an insulin-resistant state will progress to frank hyperglycemia/hyperlipidemia and diabetes; insulin resistance alone is insufficient to predict T2DM [51,52]. High circulating concentrations of glucose and fatty acids in diabetic states are attributed to loss of islet function and mass due to glucolipotoxicity, a process involving oxidative stress, ER stress, and inflammation [53]. Hence, the development of therapeutic agents that can protect islets from glucolipotoxicity could provide a much needed mode of improving the management of T2DM.

Several signaling pathways have been reported to play critical roles in insulin secretion as well as beta-cell growth and survival. For example, activation of Akt is closely associated with beta-cell survival [54,55,56] and promotes compensatory beta-cell growth in the insulin-resistant state [57]. Akt activation-induced phosphorylation inhibits GSK3 and Foxo1 which, in turn, reverses the toxic effects of glucose and fatty acids on beta-cells [58,59]. In addition, fatty acid-induced ER stress in islets has been linked with decreased Akt activity, and with activation of c-Jun NH_2_-terminal kinase (JNK), which, ultimately, contributes to beta-cell apoptosis [60]. These findings lend support to the notion that Akt is a promising target for preservation of islet function and cell mass. In this context, recent studies have demonstrated that angiotensin (1–7), an active component of the renin-angiotensin system (RAS), modulates insulin resistance in skeletal muscle cells by way of Akt signaling pathway activation [61]. Activation of Akt/JNK pathways is also involved in angiotensin (1–7)-mediated modulation of palmitate-induced islet endothelial cell apoptosis [62]. Interestingly, it has been shown that glucagon-like peptide-1 and angiotensin II can prevent glucolipotoxicity-induced apoptosis in pancreatic beta cells additively through the insulin receptor substrate-2/phosphoinostide 3-kinase/Akt/FoxO1 signaling pathways [63]. Hence, it is plausible that other regulators, such as vitamin D (*vide supra*) might, like RAS ligands, exert islet-protective effects through activation of Akt signaling. Further work is needed to examine potential vitamin D-RAS axis interactions in the regulation of islet function and beta-cell survival.

Given the importance of hepatic and pancreatic islet functions in the pathogenesis of insulin resistance and T2DM, we are investigating promising factors and how they, alone or in combination, influence islet function under various pathological and physiological conditions. Of great interest in this context is the local pancreatic RAS and its potential involvement in islet function and survival in T2DM [64]. Previously, we demonstrated that inhibition of islet RAS signaling (*i.e.*, angiotensin II receptor type 1 activation) increased glucose-induced insulin secretion and improved glycemic control [65,66]. Mechanisms proposed for these effects include enhancement of intra-islet blood perfusion [67], reduction of oxidative stress [68], and improvement of the beta-cell proliferation-to-apoptosis balance [69]. Meanwhile, vitamin D is necessary for normal islet insulin secretion [9,10,11] and it is also a negative endocrine regulator of the RAS, suppressing renal renin secretion [70,71]. Moreover, hypovitaminosis D leads to defective insulin secretion, reduced glucose homeostasis, and increased risk of T2DM in all age bands [72,73], whereas increased pancreatic islet RAS activity in hyperglycemia impairs islet function and survival under hyperglycemic conditions [74]. Given these vitamin D-RAS interactions in islet survival, we set out to examine the potential modulatory action of vitamin D on regulation of islet function and survival through suppression of pancreatic islet RAS [74,75].

Toward this aim, we examined in *ex vivo* experiments how calcitriol (bioactive vitamin D metabolite) affects RAS component expression in islets from normal control, hypovitaminosis D, and VDR knockout mice under physiological and high-glucose conditions. We found that high-glucose–induced upregulation of islet RAS component expression could be prevented and corrected by calcitriol; in corroboration, the VDR knockout mice exhibited overactive islet RAS, compared with that of wild-type mice [76]. On the other hand, the mice with diet-induced hypovitaminosis D developed impaired glucose tolerance and exhibited increased expression RAS components combined with reduced expression of islet function-related genes [76]. Interestingly, we observed in a subsequent study that pharmacological renin inhibition (*i.e.*, aliskiren treatment) without correction of hypovitaminosis D reduced islet RAS hyperactivity, ameliorated islet dysfunction and insulin resistance, and improved glucose tolerance [77].

**Figure 3:**
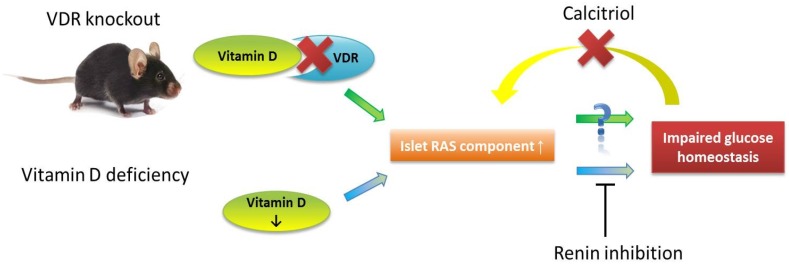
Model of vitamin D in the regulation of pancreatic islet beta-cell function and survival in a diabetic state.

In summary, treating mice with hypovitaminosis D with RAS inhibitors, without correction of vitamin D deficiency, reduced islet RAS over-activity, ameliorated islet dysfunction and improved glucose tolerance, as predicted. These data indicate that suppression of RAS hyperactivity in a condition of hyperglycemia and hypovitaminosis D is protective against pancreatic islet dysfunction and the development of insulin resistance, thus improving glucose tolerance and glucose homeostasis. If corrected, these findings help to explain the protective role of RAS inhibition against T2DM, as previously reported, and support the use of RAS inhibitors for treating hypertension in people who have, or are at risk of, T2DM. Further work is needed to determine whether alleviation of hypovitaminosis D could work synergistically with RAS inhibition to improve T2DM-related metabolic impairments. In light of these findings, we conclude with Figure 3 that represents a proposed model of how vitamin D regulates pancreatic beta-cell function and survival.

## 6. Vitamin D and Pancreatic Islet Development

Recent advances in directed differentiation of pancreatic stem cells offer potential for the development of beta-cell replacement therapies for diabetes patients. Unfortunately, however, existing differentiation protocols are complex, time-consuming, and costly; thus there is a desperate need for alternative protocols that can be used to promote beta-cell proliferation, differentiation, and maturation [78]. In this regard, our group has established a system for the isolation and culture of pancreatic progenitor cells (PPCs) derived from human fetal pancreas tissue. These PPCs have a high capacity for proliferation and differentiation when cultured with an appropriate differentiation cocktail; their differentiation can be promoted by morphogens or growth factors related to human pancreatic development, e.g., secreted PDZ domain-containing protein 2 [79,80] and angiotensin II [81], as well as by vitamin A and vitamin D [82]. Vitamin A is a well-established modulator of beta-cell differentiation, and vitamin D is known to affect beta-cell insulin secretion; both vitamin A and vitamin D act through the RXR heterodimerization pathway (*vide supra*) [83,84,85].

Interestingly, we have demonstrated that the retinoic acid receptor, VDR, and RXR are expressed in first-trimester human fetal PPCs and that all-trans retinoic acid and calcitriol can (each alone) enhance PPC viability [82]. Although further investigations are warranted to elucidate the differentiation properties of PPCs and clarify the roles of vitamin A and vitamin D in islet development and beta-cell differentiation, these data suggest that vitamin A and vitamin D are involved in PPC development and thus should be considered in attempts to develop culture protocols for the development of insulin-secreting islet-like cell clusters (ICC) suitable for clinical transplantation into diabetic patients.

Given the common developmental origins of the liver and pancreas, it is also interesting to note that PPCs can be differentiated in a culture devoid of growth factors by using a microenvironment established by liver stromal cells (LSC) derived from human fetal liver [86]. Specifically, we demonstrated experimentally that a liver stromal cell-induced niche can enhance PPC differentiation into ICCs and enhance ICC functionality. This was the first to report that an LSC-induced niche can enhance ICC differentiation and functionality. Further modifications of the stroma microenvironment may offer an alternative, efficient and cost-effective approach to providing insulin-producing cells for clinical transplantation.

Finally, very recent studies implicated vitamin D in trans-generational risk of metabolic disease. A maternal high-fat diet during pregnancy and lactation was reported to affect hepatic fatty acid metabolism of rat offspring [87]. These animal findings have clinical relevance to obesity-associated diseases in humans and future studies are warranted to examine whether specific plasma fatty acid could be used as an early marker of hepatic dysregulation so as to assist in the identification of offspring that might have a risk of increased adiposity in adulthood. In fact, hypovitaminosis D is closely related to obesity [88] and, in fact, obesity is a causal factor for reduction of serum 25(OH)D levels [89]. These findings suggest that low vitamin D availability due to excessive maternal body fat may contribute to trans-generational impairment of liver fatty acid metabolism [90].

## 7. Conclusions

Vitamin D might have dual anti-diabetic influences: (1) modulation of hepatic glucose and lipid metabolism; and (2) promotion of pancreatic islet function and survival. Vitamin D could ameliorate hepatic glucose and lipid metabolism abnormalities *in vitro* and *in vivo* through activation of Ca^2+^/CaMKKβ/AMPK signaling. Furthermore, vitamin D might have a RAS-suppressing influence that may benefit beta-cell function. Taking into consideration some negative data from clinical trial studies, our preliminary results with demonstrated beneficial effects of vitamin D on hepatic and pancreatic functions await to be intensively investigated and validated. In addition, our study findings obtained from cellular and animal models should also be interpreted cautiously since they are not always readily translated into human. However, given the high worldwide prevalence of obesity, T2DM, and related cardio-metabolic sequela that are associated with high healthcare costs and socio-economic implications, the potential protective effects of vitamin D warrants further exploration; if confirmed, vitamin D supplementation may represent a promising, cost-effective preventative and therapeutic agent for the management of obesity-related insulin resistance and diabetes.
